# Targeting NEDD8-activating enzyme for cancer therapy: developments, clinical trials, challenges and future research directions

**DOI:** 10.1186/s13045-023-01485-7

**Published:** 2023-07-31

**Authors:** Dong-Jun Fu, Ting Wang

**Affiliations:** Beijing Research Institute of Chinese Medicine, Beijing University of Chinese Medicine, Beijing, China

**Keywords:** NEDDylation, NAE, Cancer therapy, Challenges, Development directions

## Abstract

NEDDylation, a post-translational modification through three-step enzymatic cascades, plays crucial roles in the regulation of diverse biological processes. NEDD8-activating enzyme (NAE) as the only activation enzyme in the NEDDylation modification has become an attractive target to develop anticancer drugs. To date, numerous inhibitors or agonists targeting NAE have been developed. Among them, covalent NAE inhibitors such as MLN4924 and TAS4464 currently entered into clinical trials for cancer therapy, particularly for hematological tumors. This review explains the relationships between NEDDylation and cancers, structural characteristics of NAE and multistep mechanisms of NEDD8 activation by NAE. In addition, the potential approaches to discover NAE inhibitors and detailed pharmacological mechanisms of NAE inhibitors in the clinical stage are explored in depth. Importantly, we reasonably investigate the challenges of NAE inhibitors for cancer therapy and possible development directions of NAE-targeting drugs in the future.

## Background

Neuronal precursor cell-expressed developmentally down-regulated protein 8 (NEDD8) shares about 60% of the amino acids with ubiquitin, which covalently binds to substrate proteins by generating an isopeptide chain between the lysine residue of substrates and the glycine residue of NEDD8 [1–5]. NEDDylation is a biochemical process of post-translational modification that conjugates NEDD8 to substrate proteins through the successive enzymatic cascades [6–10]. In the initial stage of NEDDylation, the precursor of NEDD8 is hydrolyzed to mature NEDD8 by the precursor processing enzymes [11–14]. Next, the mature NEDD8 is activated in the presence of adenosine triphosphate (ATP) by the E1 NEDD8-activating enzyme (NAE) consisting of amyloid protein-binding protein 1 (APPBP1) and ubiquitin-like modifier activating enzyme 3 (UBA3) [15–17]. Then, the activated NEDD8 is transferred to NEDD8-conjugating enzyme E2s (UBC12 and UBE2F) via a trans-thiolation process [18–23]. Finally, NEDD8 is transferred from NEDD8-conjugating enzyme E2s to a specific lysine residue of substrates through the catalytic action of NEDD8 E3 ligases [24–26]. In recent years, homogeneous time-resolved fluorescence [27], AlphaScreen [28], in vitro NEDD8 conjugation assay [29], cellular thermal shift assay [30], co-immunoprecipitation [31], pull-down assay [32] and ATP kinetics assay [33] are commonly used to identify NEDDylation process.

The main substrates for NEDDylation pathway are members of the cullin family (cullin1, 2, 3, 4A, 4B and 5), which are the core components of Cullin-RING ligases (CRLs) [34–36]. The activation of CRLs requires NEDD8 to be conjugated to lysine residues at C-terminus of cullins, thereby inducing conformational changes in the CRLs complex and eventually regulating ubiquitylation process [37, 38]. As the most important ubiquitin ligase family in E3, CRLs significantly control a variety of basic biological functions by heightening the activity of ubiquitylation and subsequent degradation of key regulatory proteins [39]. Although cullins are well-characterized substrates in NEDDylation pathway, some non-cullins have also been identified as substrate proteins of the NEDDylation modification, such as p21, p53, p73, caspase7, XIAP, RhoA, HuR and ATF4 [40]. The characteristics of cullin and non-cullin substrates determine the significant role of NEDDylation in regulating biological processes and controlling multiple diseases, primarily neurodegenerative diseases and human cancers [41].

**Fig. 1:**
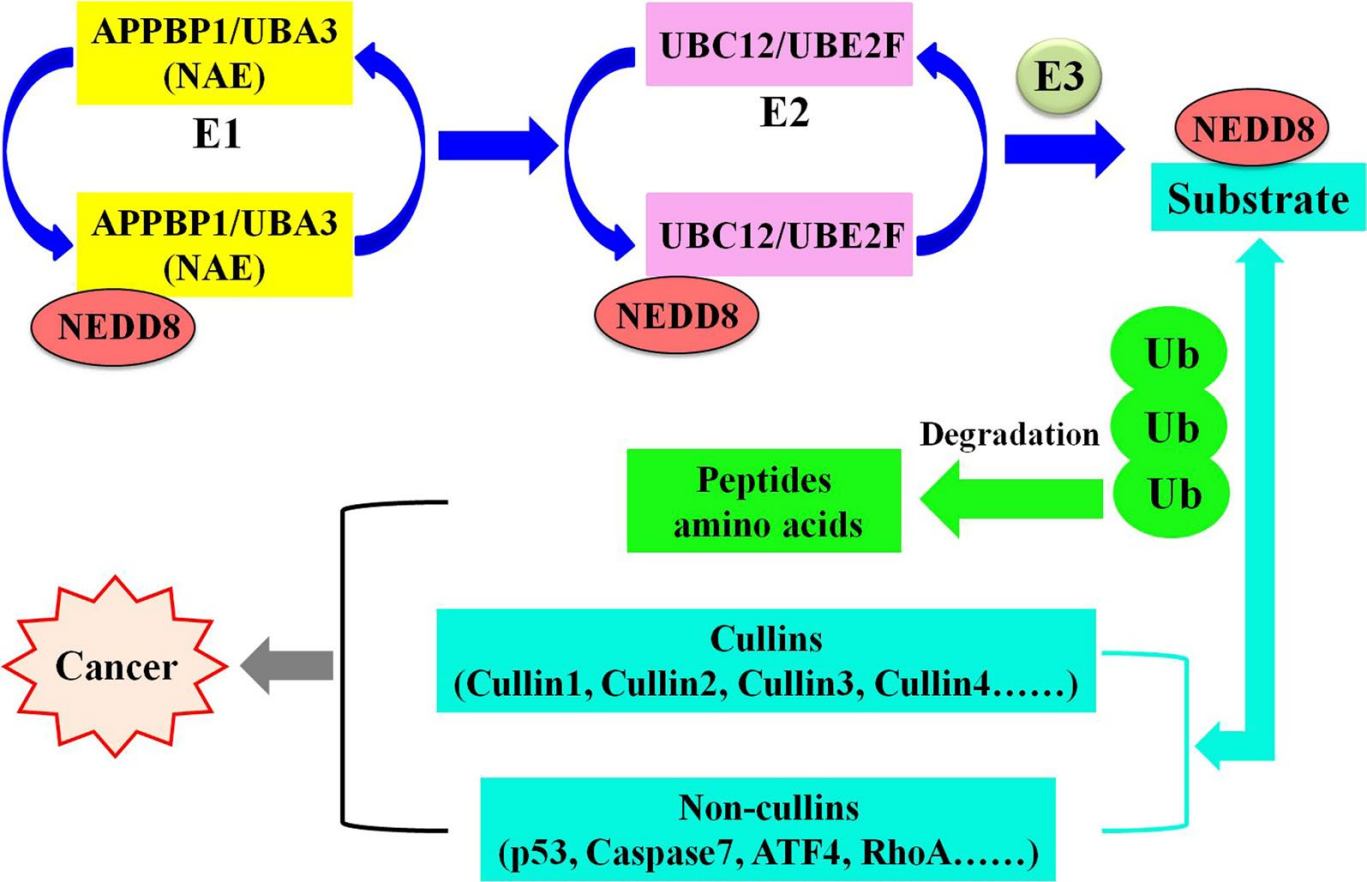
Targeting NAE as a promising therapeutic approach for cancer

The discovery of the covalent NAE inhibitor MLN4924 in 2009 set a landmark, demonstrating that targeting NEDDylation is an effective anticancer strategy [42]. The inhibition of NEDDylation by NAE inhibitors leads to the decrease of CRLs levels during ubiquitination process and ultimately results in the degradation of proteins that play significant roles in cell proliferation, DNA damage, cell cycle, stress responses and signal transduction [43–49]. In addition, targeting NAE for cancer therapy induces the abnormal NEDDylation modification of cullin and non-cullin substrates [50]. The relationships between NAE and cancers are summarized in Fig. 1. Thus, NAE as the only activation enzyme in NEDDylation process has been a promising target for the treatment of hematological tumors, solid tumors, etc. [51–55].

## Structure of NAE

**Fig. 2:**
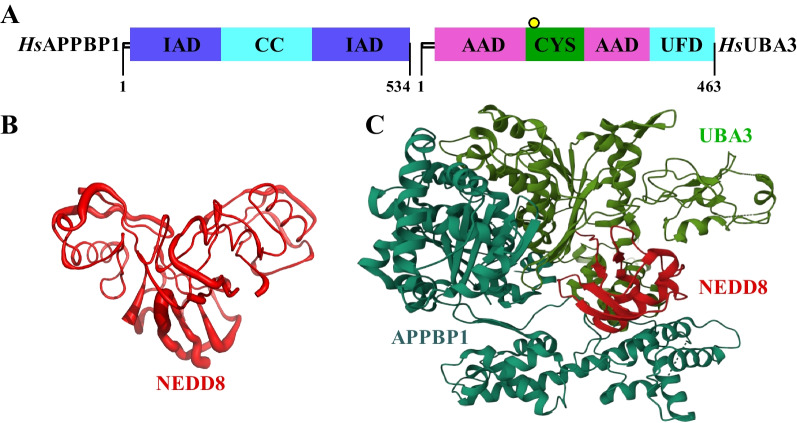
Structure of NAE and its complex. **A** Primary structure of NAE. **B** Crystal structure of NEDD8. **C** Crystal structure of NAE-NEDD8 complex; APPBP1 part is wathet blue, UBA3 part is green and NEDD8 part is red

Canonical E1 enzymes including UAE, SAE and NAE have a common structure consisting of two pseudosymmetric adenylation domains: the active adenylation domain (AAD) and the inactive adenylation domain (IAD) [56–61]. As shown in Fig. 2A, NAE contains an insertion within the IAD, which is known as the catalytic cysteine (CC) domain in APPBP1 [62]. Furthermore, NAE also contains a ubiquitin fold domain (UFD) at the C-terminal of the AAD in UBA3 and a conserved CYS domain inserted within the AAD [63–65]. In the heterodimeric NAE, APPBP1 subunit corresponds to the N-terminal of the single-chain NAE, and UBA3 as the other subunit corresponds to the C-terminal of the single-chain NAE [66–68]. The biological function of NAE is to activate NEDD8 through the formation of NEDD8-NAE thioester conjugate, thereby completing the NEDDylation process of substrate proteins [69–71]. The crystal structure of NEDD8 is shown in Fig. 2B (PDB code: 1NDD, resolution: 1.60 Å) [72]. Furthermore, the crystal structure of NAE-NEDD8 complex is shown in Fig. 2C (PDB code: 1R4M, resolution: 3.00 Å).

X-ray structures of NAE and NAE complex with the high-resolution can be available in protein data bank (https://www.rcsb.org/). In Table 1, we summarize the resolution and macromolecule contents of these X-ray structures. Total structure weight of NAE as a single enzyme is 220.58 kDa (PDB code: 1YOV, resolution: 2.60 Å). The NAE complexes, including 1R4N, 1R4M, 1TT5, 2NVU, 3DBR, 3DBL and 3DBH, demonstrate the structural basis for the activation of NEDD8 by NAE. The co-crystal structure of APPBP1/UBA3/NEDD8 with a small molecule inhibitor provides an extremely important tool for the rational design and efficient virtual screening of NAE-targeting agents (PDB code: 3GZN, resolution: 3.00 Å).

**Table 1:** X-ray structures of NAE and NAE complex

Enzyme/ligand name	PDB code	Resolution (Å)	Total structure weight (kDa)	Atom count	Modeled residue count	Deposited residue count	Unique protein chains	References
NAE(APPBP1/UBA3)	1YOV	2.60	220.58	14,604	1834	1962	2	[62]
APPBP1/UBA3/NEDD8/ATP	1R4N	3.60	469.48	31,744	4040	4144	3	[73]
APPBP1/UBA3/NEDD8	1R4M	3.00	467.45	31,620	4040	4144	3	[73]
APPBP1/UBA3/UBC12	1TT5	2.60	223.43	14,637	1866	1982	3	[74]
APPBP1/UBA3/NEDD8/ATP/UBC12	2NVU	2.80	188.89	13,090	1647	1683	4	[75]
APPBP1/UBA3/NEDD8	3DBR	3.05	474.00	32,424	4098	4212	3	[76]
APPBP1/UBA3/NEDD8	3DBL	2.90	474	32,577	4121	4212	3	[76]
APPBP1/UBA3/NEDD8	3DBH	2.85	473.77	32,578	4123	4212	3	[76]
APPBP1/UBA3/NEDD8/MLN4924	3GZN	3.00	244.27	16,050	2069	2158	3	[77]

## Multistep mechanisms of NEDD8 activation by NAE and approaches to discover NAE inhibitors

**Fig. 3:**
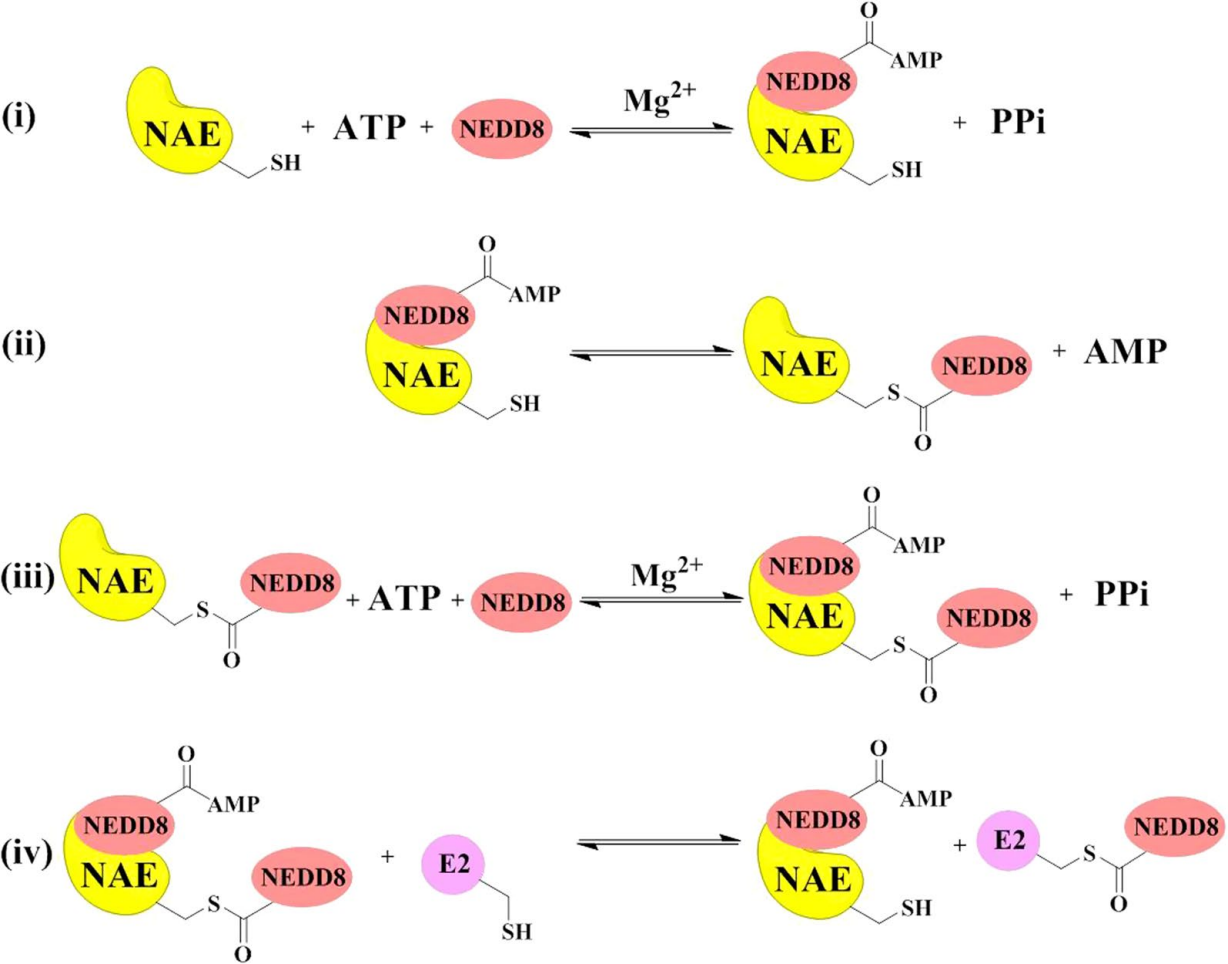
Detailed mechanisms of NEDD8 activation by NAE

The multistep mechanisms of NEDD8 activation by NAE are summarized in Fig. 3. In the first reaction (i), NAE binds NEDD8 and ATP to form a high-energy NEDD8-AMP intermediate and release PPi in the presence of magnesium [73, 76, 78]. In the second reaction (ii), the catalytic cysteine of NAE reacts with the high-energy NEDD8-AMP intermediate to form a thioester bond between NAE and NEDD8 and release AMP [79]. In the third reaction (iii), NAE-NEDD8 intermediate binds NEDD8 and ATP to catalyze formation of a NEDD8-NAE-NEDD8-AMP complex and release PPi in the presence of magnesium [80]. In the final reaction (iv), a thioester-linked E2-NEDD8 is formed from the NEDD8-NAE-NEDD8-AMP complex in the presence of E2 via a transthioesterification reaction [81].

The structural studies of NAE and detailed mechanisms of NEDD8 activation by NAE provide at least four approaches to discover potential NAE inhibitors. Approach (1) is the discovery of ATP-competitive NAE inhibitors for blocking the binding of ATP to the active adenylation site in NAE [82–84]. Approach (2) is the discovery of AMP analogues as covalent NAE inhibitors for the formation of NEDD8-compound covalent adducts to prevent the subsequent NEDD8 activating steps [42]. Approach (3) is the discovery of novel NAE inhibitors targeting the cysteine active-site for blocking the formation of NAE-NEDD8 thioesters [75]. Approach (4) is the discovery of novel NAE inhibitors disrupting NAE-E2 interactions for blocking the transferation of NEDD8 to NEDD8-conjugating enzyme E2s [74]. Most of the current NAE inhibitors are reported to exert their inhibitory effects through the first two modes. Currently, most reported inhibitors display the inhibitory activity against NAE via the first two modes, while NAE inhibitors with latter two modes have not been studied deeply.

## Preclinical studies and pharmacokinetics of covalent NAE inhibitors

Up to now, design strategies, synthetic methods and anticancer mechanisms of various NAE-targeting agents have been developed [85–89]. Compared with non-covalent NAE inhibitors and NAE agonists, developments of covalent NAE inhibitors are relatively mature. All reported covalent inhibitors contain a sulfanilamide group to form the covalent bond with NAE. Chemical structures, enzymatic activity and cellular activity of reported covalent NAE inhibitors **1**–**9** are summarized in Table 2. In 2009, MLN4924 (compound 1) as the first NAE inhibitor was reported by Millennium Pharmaceuticals, Inc [42]. MLN4924 displays a potent inhibitory activity against NAE with an IC_50_ value of 4 nM. However, it has relatively poor activity against UBA6, SAE, UAE and ATG7 with IC_50_ values of 1.8 μM, 8.2 μM, 1.5 μM and > 10 μM, respectively. The selective inhibition of NAE activity has been an effective strategy for cancer therapy, and MLN4924 as a selective NAE inhibitor exhibits anticancer effects against various cancer types, including leukemia [90], endometrial carcinoma [91], renal cell carcinoma [92], urothelial carcinoma [93], liver cancer [94], colorectal cancer [95], ovarian cancer [96], glioblastoma [97], pancreatic cancer [98], cervical cancer [99], lung cancer [100], breast cancer [101], head and neck squamous cell carcinoma [102], nasopharyngeal carcinoma [103], uveal melanoma [104], gastric cancer [105], malignant melanoma [106], retinoblastoma [107], multiple myeloma [108], prostate cancer [109], osteosarcoma [110], malignant rhabdoid tumors [111], ewing sarcoma [112] and lymphoma [113].

**Table 2:** Reported covalent NAE inhibitors

Chemical structure	Number	IC_50_ (enzymatic level)	Target	IC_50_ (cellular level)	Cell line	Year	References
	**1** (MLN4924)	4.00 nM	NAE	52.48–5552.47 nM	MV-4-11, LNCaP, COLO 205, THP-1, HGC27, SW620, etc.	2009	[42]
	**2**	2.80 μM	Pan-E1	Unknown	HCT-116	2011	[118]
	**3**	< 10.00 nM	NAE	160.00 nM	K562	2011	[119]
	**4**	< 10.00 μM	Pan-E1	Unknown	A549, LNCap, MCF7, HeLa	2013	[120]
	**5**	1.06 μM	NAE	12.30 − 29.50 μM	Caco-2, MCF-7, Bcl-7402	2014	[121]
	**6**	< 0.10 μM	NAE, UAE	2.50 μM	A549	2015	[122]
	**7** (TAS4464)	0.96 nM	NAE	1.60–460.00 nM	Patient-derived AML cells	2019	[114]
	**8**	0.55 nM	NAE	33.90–482.00 nM	HCT-116, HuTu80, Capan-1, MV-4–11, THP-1, etc.	2021	[123]
	**9**	0.36 nM	NAE	5.59–2164.76 nM	SW48, COLO 205, Capan-1, THP-1, MV-4-11, HCT-116, etc.	2022	[124]

Compared with covalent NAE inhibitors **1**–**6**, TAS4464 (compound **7**) displays more potent inhibitory activity against NAE with an IC_50_ value of 0.96 nM [114]. Moreover, TAS4464 shows inhibitory effects against SAE, UAE and carbonic anhydrase with IC_50_ values of 1280 nM, 449 nM and 730 nM, suggesting that TAS4464 is a selective NAE inhibitor. TAS4464 has potent growth-inhibitory effects against various kinds of cancers, including chronic lymphocytic leukemia, T cell acute lymphoblastic leukemia, mantle cell lymphoma, clear cell sarcoma, colon carcinoma, follicular lymphoma, small cell lung cancer, multiple myeloma, acute myeloid leukemia and diffuse large B cell lymphoma [115–117]. The antiproliferative activity of TAS4464 is 3–64 times more potent than that of MLN4924 in 240 cell lines derived from human tumor tissues. It display potent antiproliferative activity with IC_50_ values of 1.60–460.00 nM, 0.70–4223.00 nM and 0.2 nM against patient-derived AML, DLBCL and SCLC cells. Moreover, TAS4464 can obviously inhibit tumor regression in CCRF-CEM, GRANTA-519 MCL, SU-CCS-1 and LU5266 xenograft models.

More potent NAE inhibitors **8** and **9** containing a pyrimidotriazole framework are designed and synthesized via a scaffold hopping strategy in Ao Zhang’s group [123, 124]. Structure–activity relationship studies reveal that the pyrimidotriazole framework plays an important role for inhibitory effects against NAE and cancer cells. Compounds **8** and **9** significantly inhibit tumor growth in leukemia MV-4-11 and colon cancer HCT-116 xenograft models. All reported pharmacokinetic parameters of covalent NAE inhibitors are summarized in Table 3. Although TAS4464 exhibits potent anticancer effects in vitro and in vivo, its detailed pharmacokinetic data are not shown [117]. Following intravenous injection (1 mg/kg), compound **9** demonstrates a 4.46-fold increment in drug exposure with the AUC_last_ value of 2500 h·ng/mL and a 5.57-fold decrease in systemic plasma clearance with the CL value of 5.33 mL/min/kg compared with MLN4924.

**Table 3:** Pharmacokinetic parameters of reported NAE inhibitors^a^

Compound	Dose (mg/kg)	Route	*T*_1/2_ (h)	T_max_ (h)	AUC_last_ (h·ng/mL)	Vss__obs_ (mL/kg)	CL (mL/min/kg)	*C*_max_ (ng/mL)
**1** (MLN4924)	3	p.o	5.50	0.92	208	NA	NA	64
**1** (MLN4924)	1	i.v	0.71	NA	560	1783	29.70	NA
**8**	1	i.v	1.36	NA	1328	1631	16.50	NA
**9**	3	p.o	3.14	1.17	810	NA	NA	350
**9**	1	i.v	5.68	NA	2500	1582	5.33	NA

^a^p.o.: oral administration; i.v.: intravenous injection; NA: not applicable

## Molecular docking studies of NAE inhibitors in the clinical stage

**Fig. 4:**
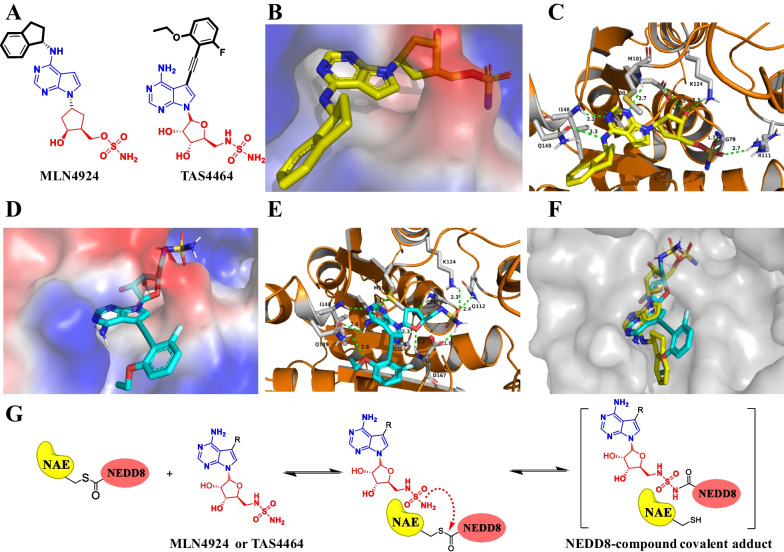
NAE inhibitors in clinical trials. **A** Chemical structures of NAE inhibitors in the clinical stage; **B** Surface map of MLN4924 in NAE (PDB: 3GZN); **C** Three-dimensional binding model of MLN4924 in NAE; **D** Surface map of TAS4464 in NAE (PDB: 3GZN); **E** Three-dimensional binding model of TAS4464 in NAE; **F** Overlay of TAS4464 (wathet blue) and MLN4924 (yellow) in complex with NAE; **G** The formation of NEDD8-compound covalent adduct

Among all reported NAE inhibitors, only MLN4924 and TAS4464 as covalent NAE inhibitors currently entered into clinical trials for cancer therapy, particularly for AML [116, 125–127]. Chemical structures of MLN4924 and TAS4464 are shown in Fig. 4A. The co-crystal structure of NAE and MLN4924 (PDB: 3GZN, resolution: 3.00 Å) was reported in 2010 and shown in Fig. 4B, C [77]. G79 and R111 of NAE form a hydrogen bond with the sulfonamide group of MLN4924 (1.7 Å and 2.7 Å). The hydroxyl group on the ribose unit generates two hydrogen bonds with K124 (2.3 Å) and D100 (3.5 Å), respectively. The nitrogen atom of secondary amine group produces a hydrogen bond with Q149 (3.3 Å), and 7*H*-pyrrolo[2,3-*d*]pyrimidine unit forms two hydrogen bonds with I148 (2.2 Å) and D100 (2.7 Å). The most important binding force for the inhibitory effect is the covalent bond between the carbonyl carbon of G76 and the sulfonamide group of MLN4924.

Molecular docking studies of TAS4464 are also investigated and shown in Fig. 4D, E (PDB: 3GZN, resolution: 3.00 Å). The sulfonamide group of TAS4464 generates three hydrogen bonds with D167 (1.9 Å), K124 (2.3 Å) and Q112 (2.4 Å). The tetrahydrofuran unit also forms three hydrogen bonds with G78 (3.0 Å), G165 (2.3 Å) and D167 (2.2 Å). The amino group attaching 7*H*-pyrrolo[2,3-*d*]pyrimidine produces two hydrogen bonds with Q149 (2.6 Å) and M101 (3.1 Å), and 7*H*-pyrrolo[2,3-*d*]pyrimidine fragment forms a hydrogen bond with I148 (2.2 Å). The oxygen atom between ethyl group and phenyl group forms a hydrogen bond with Q149 (2.8 Å). As shown in Fig. 4F, TAS4464 occupies the same active pocket of NAE with MLN4924 and takes a similar binding pose. MLN4924 and TAS4464, which have a similar structure to adenosine 5'-monophosphate (AMP) and attack the thioester bond of NAE-NEDD8 intermediate to form NEDD8-compound covalent adducts for blocking the subsequent NEDD8 activating processes (Fig. 4G).

## Pharmacological mechanisms of MLN4924 in cancers

### Induction of apoptosis

Targeting NAE with MLN4924 induces the intrinsic apoptosis and extrinsic apoptosis through the inactivation of cullin NEDDylation [128–132]. The expression levels of cleaved caspase3 and cleaved caspase7 are increased, and the expression levels of Bcl-2 and Bcl-XL are decreased in a concentration-dependent manner with the treatment of MLN4924 against different cancer cells. Apoptosis induced by pharmaceutical inhibition of NEDDylation suppresses tumor growth in leukemia [133–135], colorectal cancer [136], ewing sarcoma [137], urothelial carcinoma [138], head and neck squamous cell carcinoma and intrahepatic cholangiocarcinoma [128, 139]. MLN4924-induced apoptosis includes enhancing the accumulation of CDT1 [140], inducing the dramatic accumulation of CRL E3 substrate I-kappa-B-alpha (IKB-α) [141] and increasing the expression of NOXA [142]. The NOXA-dependent apoptosis triggered by MLN4924 is activated by the elevated expression levels of c-Myc and transcription factor ATF-4. The combination of TNF-related apoptosis-inducing ligand (TRAIL) and MLN4924 synergistically causes apoptosis against head and neck squamous cell carcinoma (HNSCC) cells through enhancing the degradation of c-FLIP [143].

### Alterations in mitochondrial functions and energy metabolism

MLN4924 generates mitochondrial fission-to-fusion conversion by the inactivation of SCF^β-TrCP^ E3 ligase and the prevention of mitochondrial translocation against breast cancer cells [144–147]. MLN4924 changes mitochondrial shape in breast cancer cells, lung adenocarcinoma cells and bronchial epithelial cells. MFN1, MFN2 and the ectopic expression of DRP1 play dominant roles in MLN4924-induced mitochondrial fission-to-fusion conversion. The inhibition of SCF^β-TrCP^ E3 ligase promotes the polyubiquitylation and subsequent accumulation of MFN1. The effects of NEDDylation blockage by MLN4924 on cell metabolism are investigated by a mass spectrometry-based metabolic profiling, and MLN4924 perturbs metabolites of mitochondrial tricarboxylic acid cycle (TCA) and glycolysis. The decrease of ATP production, the reduction of mitochondrial depolarization and an increase of mitochondrial ROS levels are induced by MLN4924 in concentration-dependent and time-dependent manners. The combined treatment of MLN4924 with inhibitors of glycolysis or mitochondrial oxidative phosphorylation system (OXPHOS) can remarkably enhance antitumor effects in vitro and in vivo.

### Increase of G2 cell cycle arrest, induction of DNA damage and inducement of radiosensitization

MLN4924 sensitizes resistant cancer cells to ionizing radiation, which demonstrates that MLN4924 can be used as a novel radiosensitizing agent [102, 148–151]. The accumulation of p21/p27/WEE1 as three substrates of CRL induces the G2 cell cycle arrest against cancer cells with the treatment of MLN4924, and the co-treatment of MLN4924 with radiation enhances the induction of G2 cell cycle by the increased accumulation of p21/p27/WEE1 [152–156]. The turnover of p21/p27/WEE1 can be obviously delayed in prostate cancer PC3 and Du145 cells with the treatment of MLN4924-radiation. MLN4924 increases the expression levels of γ-H2AX and p-H2AX in a concentration-dependent manner, suggesting that MLN4924 causes DNA damage [157]. MLN4924-radiation combination also enhances the induction of DNA damage via the increased accumulation of ORC1 and CDT1 as CRL substrates. Radiosensitization by MLN4924 is attributable to the increase of G2 cell cycle arrest and induction of DNA damage. With the combination of radiation and MLN4924, antitumor effects in xenograft tumor models are remarkably enhanced.

### Activation of autophagy

Autophagy as a lysosomal degradation process has significant roles in the development and metastasis of cancers [158–163]. MLN4924 triggers the conversion of LC3-I to LC3-II and up-regulates the accumulation of LC3-II in a time-dependent manner, indicating that it activates autophagy [164–167]. With the treatment of MLN4924, the expression level of DEPTOR as a substrate of SCF^βTrCP^ E3 is increased in a cell line-dependent manner. MLN4924 concentration-dependently inactivates Cullin2 NEDDylation and subsequently induces a significant increase of HIF1α as a substrate of Cullin2. HIF1α-REDD1-TSC1 axis and DEPTOR are effectively involved in MLN4924-mediated mTORC1 suppression and autophagy inducement. Specifically, HIF1α triggers REDD1-TSC1 signal axis to suppress mTORC1 and DEPTOR directly binds to mTORC1, resulting in the activation of autophagy. Bafilomycin A1 as a well-known inhibitor of autophagy remarkably increases MLN4924-induced apoptosis by the up-regulated expression levels of cleaved PARP and cleaved caspase3/7. This combination of an autophagy inhibitor and a NAE inhibitor effectively increases the inhibitory effects of tumor growth through the strengthening of apoptosis.

### Trigger of senescence

Cellular senescence is an irreversible arrest of cell growth, and bioactive compound targeting tumor cell senescence has been an effective strategy for cancer therapy [168–172]. MLN4924 induces cellular senescence by inactivating CRL/SCF E3s and thus exerts antitumor effects in vivo and in vitro against osteosarcoma, colorectal cancer, lung cancer, glioblastoma, gastric cancer, lymphoma, melanoma and laryngeal cancer [173–177]. MLN4924 triggers cellular senescence in p53-null H1299 cells, indicating that MLN4924-induced senescence is not related to p53. MLN4924 increases the expression level of p21 in cancer cells, and p21 as a substrate of CRL/SCF is responsible for MLN4924-induced senescence. The senescence-like morphology induced by MLN4924 is not changed after the removal of this drug, demonstrating that the trigger of senescence by MLN4924 is irreversible.

### Suppression of tumor angiogenesis

The development of new blood vessels is called angiogenesis [178–181], and tumor angiogenesis is a potential target for the treatment of cancer [182–184]. With the intervention of MLN4924 in human microvascular endothelial cells, the branch count, junction count, area and skeleton length of the vascular network are obviously reduced [185]. MLN4924 potently suppresses the growth of new blood vessel in the rat aortic ring assay, significantly inhibits the formation of capillary vessels in the chick embryo chorioallantoic membrane assay and strongly reduces the density of microvessel in the matrigel plug assay [186]. These results from above angiogenic assays suggest that MLN4924 displays remarkably inhibitory effects on tumor angiogenesis. Importantly, MLN4924 induces the suppression of tumor angiogenesis in footpad xenograft and orthotopic models bearing pancreatic cancer cells. MLN4924 up-regulates the expression level of RhoA, which is a substrate of CRL E3. Knockdown of RhoA obviously restores the inhibitory effects of MLN4924 on the formation of capillary tube in human umbilical vein endothelial cells. Many studies demonstrate that induction of apoptosis and cell cycle arrest by MLN4924 can also suppress tumor angiogenesis [187–189]. In uveal melanoma cells, MLN4924 exerts antiangiogenic effects by impairing the secretion of VEGF-C [104].

### Regulation of tumor microenvironment

**Fig. 5:**
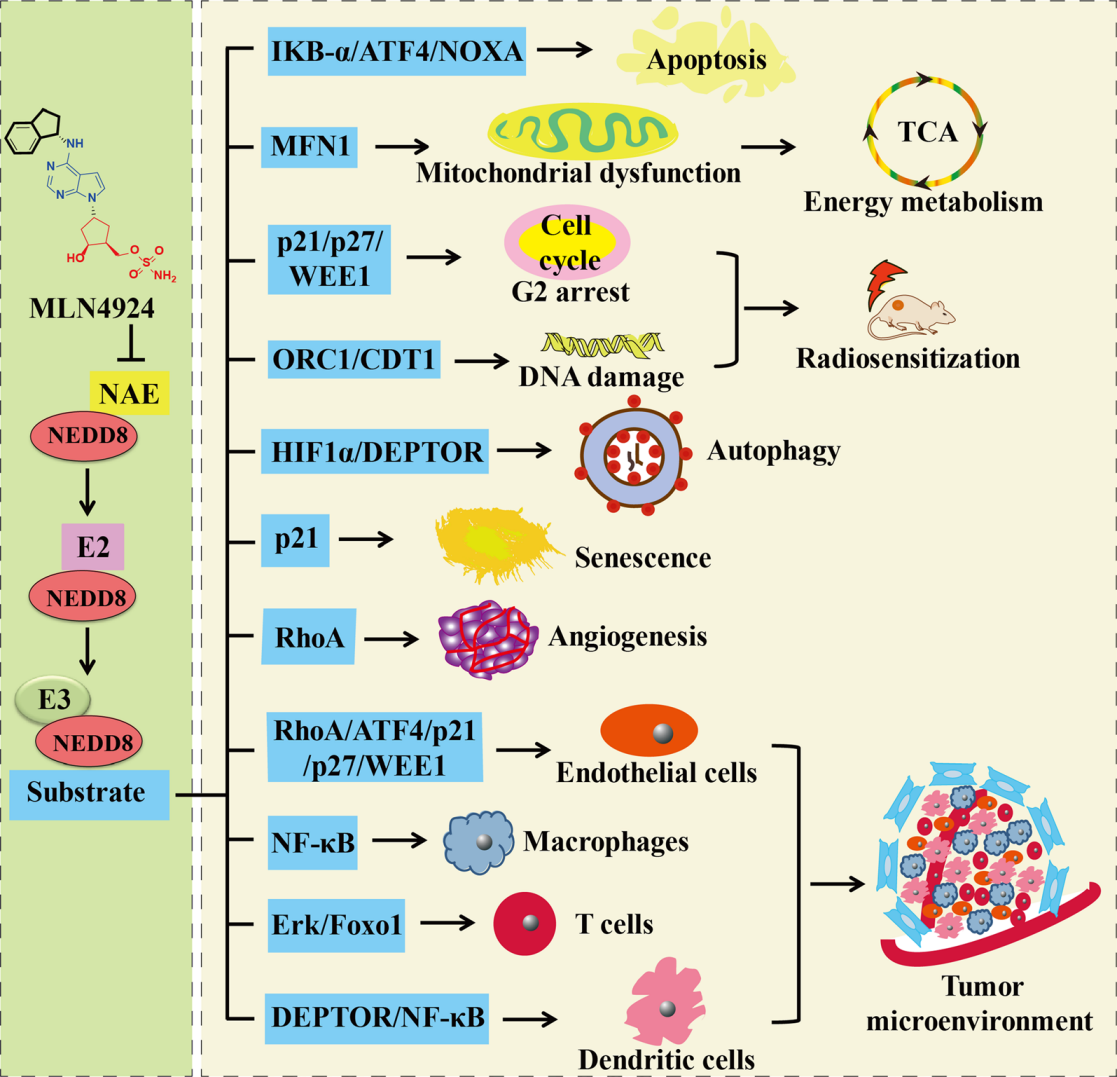
Pharmacological mechanisms of MLN4924 in cancers

Due to important roles of the tumor microenvironment (TME) in dynamically regulating tumor progression, therapeutic strategy targeting TME has become an attractive approach for cancer therapy [190–193]. The TME is the complicated multicellular environment that mainly comprises tumor-derived factors, cancer-associated fibroblasts, tumor-associated endothelial cells, natural killer cells, dendritic cells, T lymphocytes, tumor-associated macrophages and the extracellular matrix [194]. Inactivation of NEDDylation by MLN4924 affects the functions of several significant components of the TME [195]. MLN4924 activates the accumulation of the migration-related substrate RhoA, apoptosis-related substrates ATF4/NOXA and cell cycle-related substrates p21/p27/WEE1 in endothelial cells. MLN4924 regulates differentially expressed genes in fibroblasts isolated from hepatocellular carcinoma tissues, and these genes are involved in DNA replication, cell cycle, cytokine–cytokine receptor interaction and TNF signaling pathway. MLN4924 at 100 nM or 500 nM decreases the transcriptional activity of nuclear factor κB (NF-κB) induced by lipopolysaccharides in macrophage cells [196]. The activation of Erk is reduced in CD4^+^ T cells with the treatment of MLN4924 at 100 nM for 16 h [197]. NEDDylation accelerates the differentiation of follicular helper T cells through modulation the degradation of Foxo1 [198]. In dendritic cells, MLN4924 triggers the accumulation of DEPTOR and inhibits the activation of NF-κB [199, 200]. Therefore, MLN4924 exerts significantly antitumor effects in a variety of cancer models by regulating apoptosis, mitochondrial fission-to-fusion conversion, cell cycle, DNA damage, radiosensitization, autophagy, senescence, angiogenesis and tumor microenvironment (Fig. 5).

## Pharmacological mechanisms of TAS4464 in cancers

### Induction of apoptosis and sub-G1 cell cycle arrest

TAS4464 decreases the expression level of Cullin-NEDD8 and increases the expression levels of CRL substrate proteins in a concentration-dependent and time-dependent manner [115]. TAS4464 increases expression levels of cleaved caspase 2/3/6/7/8/9/10 and cleaved PARP in leukemia HL60 and THP1 cell lines, demonstrating that TAS4464 can activate extrinsic and intrinsic apoptotic pathways [116]. With the treatment of TAS4464 at 100 nM in HL60 and THP1 cells, the mRNA transcriptional level of NOXA is increased and the mRNA transcriptional level of c-FLIP is decreased. In human AML xenograft models, TAS4464 at 100 mg kg^−1^ per day remarkably inhibits cancer cell growth through the increased expression level of NOXA and the decreased expression level of c-FLIP accompanied by the activation of c-Myc. TAS4464 induces the accumulation of p21/p27/CDT1 in concentration-dependent and time-dependent manners and subsequently arrests cell cycle at sub-G1 phase.

### Inactivation of NF-κB pathways

**Fig. 6:**
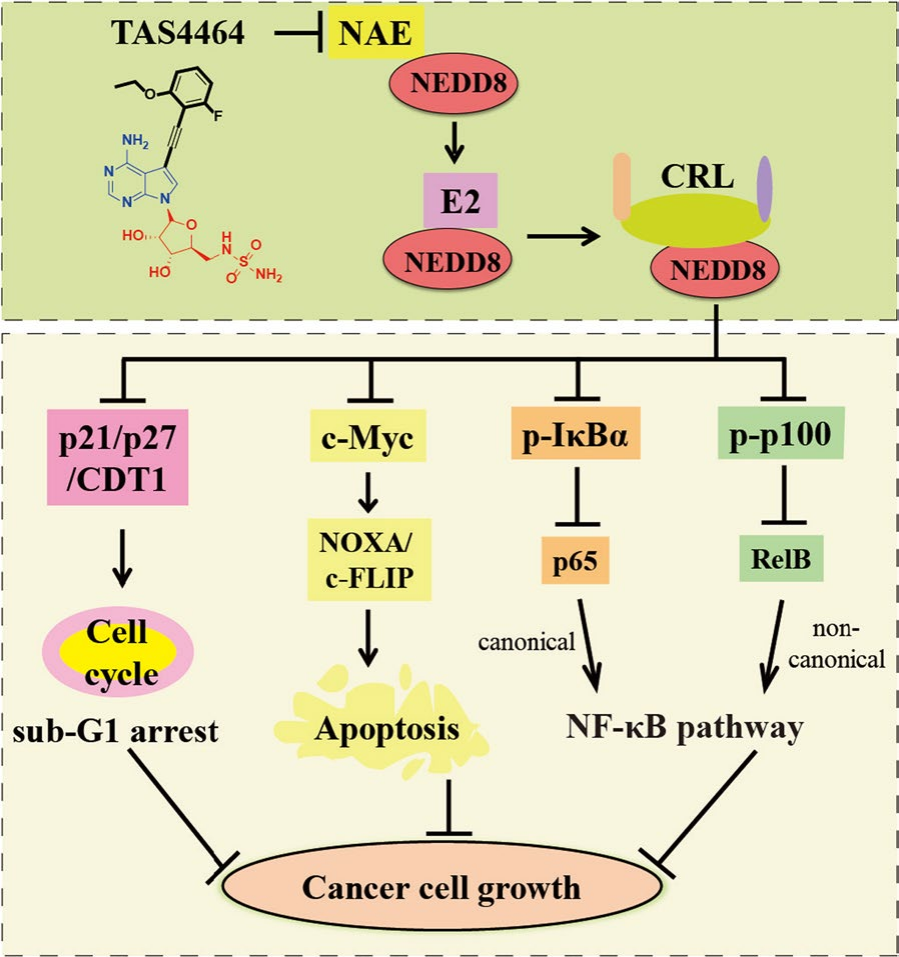
Pharmacological mechanisms of TAS4464 in cancers

With the treatment of TAS4464 for 4 h against myeloma KMS-11, MM.1S, KMS-26 and KMS-12-BM cells, the expression levels of NF-κB regulators p-p100 and p-IκBα are increased [115]. The DNA binding activity of RelB and p65 in KMS-26 and MM.1S cells is inhibited by TAS4464 in a concentration-dependent manner. Furthermore, TAS4464 suppresses the transcription levels of NF-κB-targeted genes in these myeloma cells. In xenograft mouse models bearing myeloma cells, TAS4464 down-regulates the NF-κB pathways and increases the expression levels of several apoptosis-related factors (cleaved caspase 3/8 and cleaved PARP). With the combination of chemotherapy drugs, TAS4464 synergistically improves the antitumor effects against multiple myeloma. The pharmacological mechanisms of TAS4464 regarding its therapeutic efficacy in cancers are shown in Fig. 6.

## Clinical trials of NAE inhibitors for cancer therapy

To date, 42 clinical trials for MLN4924 and TAS4464 have been registered (Table 4). Among them, five clinical trials at phase I of MLN4924 in patients with AML (ClinicalTrials.gov Identifier: NCT00911066, NCT03459859, NCT02610777, NCT02782468 and NCT03814005) have been completed [201–207]. For elderly patients with AML considered unfit for conventional chemotherapy, the combination therapy with MLN4924 and azacitidine was generally well tolerated (ClinicalTrials.gov Identifier: NCT01814826) [208]. In addition, six clinical trials at phase I of MLN4924 in patients with nonhematologic malignancies or solid tumors (ClinicalTrials.gov Identifier: NCT00677170, NCT01862328, NCT02122770, NCT03057366, NCT03330106 and NCT03486314) have also been completed [209–212]. For pediatric patients with recurrent or refractory solid tumors, MLN4924 in combination with temozolomide and irinotecan is well tolerated (ClinicalTrials.gov Identifier: NCT03323034) [213]. In this clinical trial, a dose of 25 mg/m^2^ dose on day 8 had a half-life of 5–6 h and a mean clearance of 20 L/h/m^2^.

**Table 4:** Overview of NAE inhibitors in clinical trials

Drugs	With combination	Phase	Trial number	Cancer	Year (first posted)	Patients number and sponsor	Intervention model	Status
MLN4924	Alone	I	NCT00677170	Nonhematologic Malignancies	2008	62; Millennium Pharmaceuticals, Inc	Single group assignment	Completed
	Alone	I	NCT00722488	MM, HM, HL, Lymphoma	2008	56; Millennium Pharmaceuticals, Inc	Single group assignment	Completed
	Azacitidine	I	NCT00911066	AML, MDS, ALL	2009	72; Millennium Pharmaceuticals, Inc	Single group assignment	Completed
	Alone	I	NCT01011530	Metastatic melanoma	2009	37; Millennium Pharmaceuticals, Inc	Single group assignment	Completed
	Azacitidine	I	NCT01814826	AML	2013	64; Millennium Pharmaceuticals, Inc	Sequential Assignment	Completed
	Docetaxel, Gemcitabine, Carboplatin, Paclitaxel	I	NCT01862328	Solid tumor	2013	64; Millennium Pharmaceuticals, Inc	Sequential assignment	Completed
	Fluconazole, Itraconazole, Docetaxel, Carboplatin, Paclitaxel	I	NCT02122770	Solid tumor	2014	51; Millennium Pharmaceuticals, Inc	Parallel assignment	Completed
	Docetaxel, Carboplatin, Paclitaxel	I	NCT03057366	Solid tumor	2017	8; Millennium Pharmaceuticals, Inc	Sequential assignment	Completed
	Temozolomide, irinotecan	I	NCT03323034	Recurrent or refractory solid tumors or lymphoma	2017	30; Children's Oncology Group	Single group assignment	Active, not recruiting
	Docetaxel, Carboplatin, Paclitaxel	I	NCT03330106	Advanced solid neoplasm	2017	68; Millennium Pharmaceuticals, Inc	Crossover assignment	Completed
	Rifampin, Docetaxel, Carboplatin, Paclitaxel	I	NCT03486314	Advanced solid neoplasm	2018	20; Millennium Pharmaceuticals, Inc	Sequential assignment	Completed
	Cytarabine	I	NCT03459859	AML and MDS	2018	12; Justin Watts, MD	Parallel assignment	Completed
	Ruxolitinib	I	NCT03386214	Myelofibrosis	2017	8; Washington University School of Medicine	Sequential assignment	Terminated
	Ibrutinib	I	NCT03479268	CLL or Non-HL	2018	18; City of Hope Medical Center	Single group assignment	Active, not recruiting
	Decitabine	I	NCT03009240	AML	2017	30; City of Hope Medical Center	Single group assignment	Active, not recruiting
	Vincristine, Dexamethasome, PEG-asparaginase, Doxorubicin, Cytarabine, Methotrexate, Hydrocortisone	I	NCT03349281	ALL	2017	6; Julio Barredo, MD	Parallel assignment	Completed
	Azacitidine	I	NCT02782468	Leukemia, Myeloid, MS	2016	23; Millennium Pharmaceuticals, Inc	Parallel assignment	Completed
	Belinostat	I	NCT03772925	AML, MDS	2018	30; National Cancer Institute	Single group assignment	Active, not recruiting
	Azacitidine	I	NCT03814005	MDS, CMML and AML	2019	17; Takeda	Sequential assignment	Completed
	Ixazomib citrate	I	NCT03770260	MM	2018	8; National Cancer Institute	Single group assignment	Active, not recruiting
	Azacitidine, Fludarabine, Phosphate, Cytarabine	I	NCT03813147	AML, MDS	2019	12; National Cancer Institute	Single group assignment	Active, not recruiting
	Azacitidine (or Decitabine) and Venetoclax	I	NCT04172844	AML	2019	24; Medical College of Wisconsin	Sequential assignment	Active, not recruiting
	Azacitidine	II	NCT02610777	MDS, CMML and AML	2015	120; Millennium Pharmaceuticals, Inc	Parallel assignment	Completed
	Docetaxel	II	NCT03228186	Non-small cell lung cancer	2017	31; University of Michigan Rogel Cancer Center	Single group assignment	Terminated
	Azacitidine	II	NCT03238248	MDS, MPN	2017	71; Vanderbilt-Ingram Cancer Center	Single group assignment	Active, not recruiting
	Azacitidine	II	NCT03745352	Relapsed or Refractory AML	2018	0; National Cancer Institute	Parallel assignment	Withdrawn
	Azacitidine	II	NCT03709576	Non-Remission AML	2018	3; Milton S. Hershey Medical Center	Single group assignment	Terminated
	Carboplatin, Paclitaxel	II	NCT04175912	Bile duct cancer	2019	52; National Cancer Institute	Parallel assignment	Active, not recruiting
	Carboplatin, Paclitaxel	II	NCT03965689	Advanced non-small cell lung cancer	2019	24; National Cancer Institute	Single group assignment	Active, not recruiting
	Azacitidine	Unknown	NCT04484363	MDS	2020	Intermediate-size population; Takeda	Unknown	No longer available
	Pembrolizumab	I/II	NCT04800627	Locally advanced unresectable solid tumor	2021	2; M.D. Anderson Cancer Center	Single group assignment	Terminated
	Decitabine, Cedazuridine	II	NCT04985656	MDS	2021	0; Takeda	Single group assignment	Withdrawn
	Azacitidine	II	NCT04712942	AML, MDS	2021	14; University of Leipzig	Parallel assignment	Completed
	Venetoclax, Azacitidine	II	NCT04266795	AML in adults unable to receive intensive chemotherapy	2020	164; Takeda	Parallel assignment	Active, not recruiting
	Azacitidine, Venetoclax	I/II	NCT03862157	Newly diagnosed AML	2019	51; M.D. Anderson Cancer Center	Single group assignment	Active, not recruiting
	Cytarabine, Idarubicin	I/II	NCT03330821	AML	2017	53; University of Southern California	Single group assignment	Active, not recruiting
	Etoposide, Prednisone, Vincristine, Cyclophosphamide, Doxorubicin, Rituximab, Filgrastim	I/II	NCT01415765	Lymphoma	2011	0; National Cancer Institute	Single group assignment	Withdrawn
	Pemetrexed	I/II	NCT03319537	Mesotheliomas	2017	9; Memorial Sloan Kettering Cancer Center	Sequential assignment	Active, not recruiting
	Azacitidine	III	NCT03268954	MDS, CMML and AML	2017	454; Takeda	Parallel assignment	Active, not recruiting
	Azacitidine	III	NCT04090736	AML not eligible for standard chemotherapy	2019	302; PETHEMA Foundation	Parallel assignment	Active, not recruiting
TAS4464	Alone	I	NCT02978235	MM or lymphoma	2016	11; Taiho Oncology, Inc	Single group assignment	Terminated
	Alone	I	JapicCTI-173,488	Advanced/metastatic solid tumors	2017	Unknown; Japan Pharmaceutical Information Center	Unknown	Unknown

Because of favorable results from phase I clinical trials for MLN4924, 15 clinical trials at phase II (ClinicalTrials.gov Identifier: NCT02610777, NCT03228186, NCT03238248, NCT03745352, NCT03709576, NCT04175912, NCT03965689, NCT04800627, NCT04985656, NCT04712942, NCT04266795, NCT03862157, NCT03330821, NCT01415765 and NCT03319537) and 2 clinical trials at phase III (ClinicalTrials.gov Identifier: NCT03268954 and NCT04090736) have been conducted. A phase I/II clinical trial of MLN4924 in combination with cytarabine and idarubicin was performed to investigate the therapeutic effects for patients with AML (ClinicalTrials.gov Identifier: NCT03330821). A phase II trial was first posted in 2019 to study the overall response rate, overall survival, progression-free survival and safety profile of MLN4924 in combination with paclitaxel and carboplatin for patients with advanced non-small cell lung cancer (ClinicalTrials.gov Identifier: NCT03965689). A phase III study was first posted in 2019 and last update posted in 2022 to compare the difference in overall survival between azacitidine alone and azacitidine plus MLN4924 in patients with AML (ClinicalTrials.gov Identifier: NCT04090736) [214].

TAS4464 is a potent and selective NAE inhibitor with broad-spectrum antitumor activity against a variety of cancer cell lines [117]. A phase I clinical trial of TAS4464 was conducted in 2016 to study the appropriate dose, pharmacogenomics, pharmacokinetics, pharmacodynamics and safety of TAS4464 for patients with multiple myeloma or lymphoma (ClinicalTrials.gov Identifier: NCT02978235). However, this clinical trial of TAS4464 was terminated in 2021 due to cases of drug-induced liver injury. In 2021, a phase I clinical trial was performed to explore the safety, clinical response, pharmacokinetics, pharmacogenomics and pharmacodynamics of TAS4464 in patients with advanced/metastatic solid tumors (Trial number: JapicCTI-173,488). The most common treatment-related adverse events in this trail are the increase of alanine aminotransferase (68.8%), the increase of aspartate aminotransferase (62.5%) and nausea (43.8%).

## Challenges of NAE inhibitors for cancer therapy

### Low response rate for AML/MDS and hepatotoxicity

Although the developments of NAE inhibitors have achieved some positive results, NAE inhibitors also face several expected or unexpected challenges. A phase I clinical trial of MLN4924 in patients with AML and myelodysplastic syndromes (MDS) has been successfully completed (ClinicalTrials.gov Identifier: NCT00911066). However, MLN4924 as a monotherapy in this trail only displayed an overall response rate of 17% [215]. In order to improve the efficacy, the structural optimization or the combined pharmacotherapy of MLN4924 is investigated. In 2021, Zhang's group performed a structural optimization of MLN4924 by a scaffold hopping strategy to design a pyrimidotriazole derivative with improved overall PK properties and good safety in vivo [123]. Although TAS4464 is a selective NAE inhibitor with potent antitumor effects, it generates drug-induced liver injury for patients with multiple myeloma or lymphoma in the phase I trial (ClinicalTrials.gov Identifier: NCT02978235). Thus, it is necessary to design novel NAE inhibitors with high potency and low hepatotoxicity.

### Resistance

In 2011, research demonstrated that the loss of phosphatase and tension homolog on chromosome ten (PTEN) in melanoma cells is a driver of drug resistance [216]. Similarly, protein levels of PTEN are decreased by 30-40% in breast cancer and the absence of PTEN also contributes to MLN4924 resistance in breast cancer cells [217]. With the absence of PTEN, the antitumor effects of MLN4924 in breast cancer cells are significantly decreased. The activity of Akt pathway is positively correlated with NEDDylation pathway in patients with high PTEN expression, but not in patients with low PTEN expression. PTEN plays an indispensable role for supression of PI3K/Akt pathway by MLN4924 in breast cancer cells.

### Sphere formation and ciliogenesis

Recently, Sun’s group reported that MLN4924 has the side effects of tumor sphere formation and ciliogenesis inhibition through the dimerization of EGFR [218, 219]. MLN4924 at 0.1 μM significantly stimulates the tumorigenesis of nude mice bearing H1299 cells and enhances the activation of EGFR and the downstream RAS/RAF/MEK/ERK signaling pathways. Further mechanistic studies demonstrate that MLN4924 does not directly bind to EGFR but instigates the dimerization of EGFR in a time-dependent manner. MLN4924 at 0.3 μM obviously inhibits cilia initiation and effectively promotes cilia disassembly in BEAS2B cells, and MLN4924 at 30 mg/kg suppresses hair re-growth in C57BL/6 mice. Protein kinase B (AKT1) plays a key role in the regulation of cilia initiation and disassembly induced by MLN4924. Although these studies raise some concerns for cancer therapy, they also provide an opportunity for future development of MLN4924 as a potential treatment for patients with abnormal ciliogenesis.

### Glycolysis

**Fig. 7:**
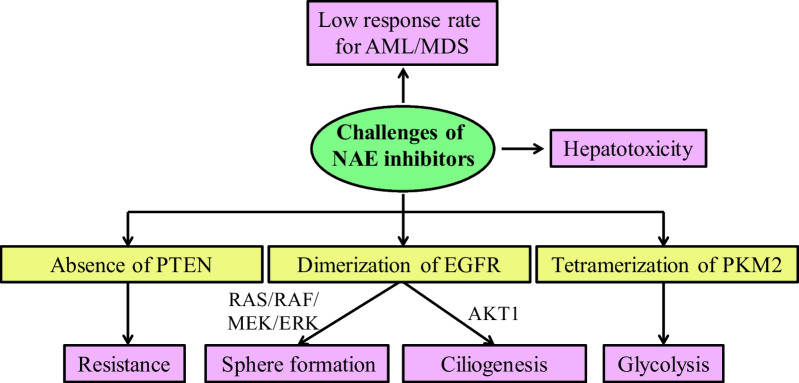
The challenges and related mechanisms of NAE inhibitors

Pyruvate kinase isoform M2 (PKM2) exhibits significant roles in cancer cell growth and metabolic reprogramming, and it is overexpressed in cancer cells to catalyze glycolysis [220]. MLN4924 promotes glycolysis through the up-regulation of PKM2 tetramers levels in a concentration-dependent manner against breast cancer MDA-MB-231 cells. Glycolysis triggered by MLN4924 confers survival to breast cancer cells, and 2-deoxy-D-glucose as a glycolysis inhibitor coupled with MLN4924 can exhibit much better effects of growth inhibition against breast cancer cells in xenograft models [144]. Therefore, the tetramerization of PKM2 to increase glycolysis and cancer cell growth has been a side effect of MLN4924 to treat breast cancer [221]. The challenges and related mechanisms of NAE inhibitors are summarized in Fig. 7.

## Potential development directions of NAE-targeting drugs in the future

### Combined pharmacotherapy or multi-target drugs

**Fig. 8:**
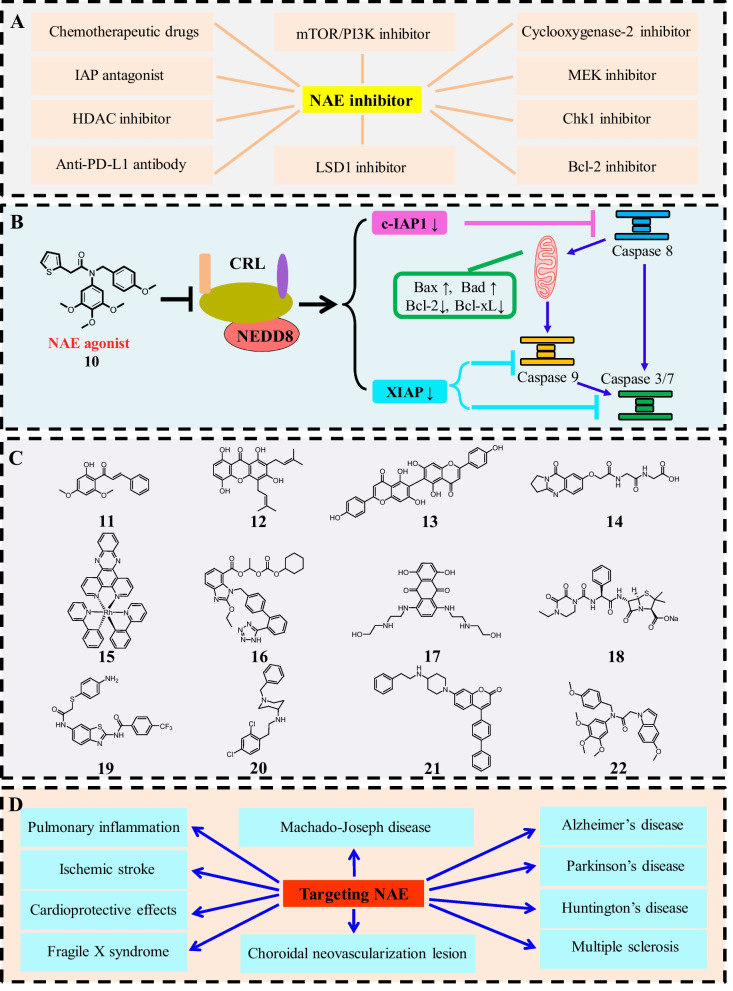
Potential future directions of NAE-targeting drugs. **A** Combination strategies with NAE inhibitor for cancer therapy; **B** NAE agonist and its anticancer mechanisms; **C** Non-covalent NAE inhibitors; **D** Application for other diseases beyond cancer by targeting NAE

Although a number of NAE inhibitors have been developed, only AMP analogues MLN4924 and TAS4464 have formally entered the clinic trails to treat various cancers. Some NAE inhibitors demonstrate moderate antitumor effects, potential hepatotoxicity and resistance against cancer cells in vitro and in vivo. In order to improve antitumor potency and reduce side effects, development of combined pharmacotherapy or multi-target drugs might be promising approaches in the future directions [222–225]. Because of the complex pathogenesis of cancer, combined pharmacotherapy can synergistically exhibit better therapeutic effects by intervening in multiple targets or signal pathways in cancer cells [226–228]. Furthermore, combined pharmacotherapy or multi-target drugs can reduce the toxic side effects and decrease the therapeutic dose [229]. To date, there is growing evidence that MLN4924 coupled with different anticancer drugs can also overcome drug resistance and improve anticancer effects [230–233]. Up to now, there are more than 30 clinical trials investigating the combined pharmacotherapy of MLN4924. Recent studies investigated that NAE inhibitor MLN4924 and HDAC inhibitor belinostat could interact synergistically by reciprocally disabling the DNA damage response in AML/MDS cells [234, 235]. Guo’s group reported that the combinational of celecoxib as a cyclooxygenase-2 inhibitor with MLN4924 synergistically inhibits the survival of human urothelial carcinoma cells by decreasing phosphorylation of AKT/ERK signaling pathways [236]. Additionally, combined pharmacotherapy of MLN4924 could enhance the antitumor activity with Chk1 inhibitor, Bcl-2 inhibitor, IAP antagonist, mTOR/PI3K inhibitor, MEK inhibitor, anti-PD-L1 antibody and LSD1 inhibitor (Fig. 8A) [237–242]. Compared with combined pharmacotherapy, multi-target drugs have therapeutic advantages due to their abilities to reduce the complexity of pharmacokinetics and drug-drug interactions [243–245]. More importantly, the successful combined pharmacotherapy in clinical trials might provide a solid experimental basis for the development of multi-target NAE inhibitors to treat cancer in the future.

### NAE agonists

In 2020, we reported the world's first NAE agonist (compound **10**) with IC_50_ values of 0.09 μM, 0.10 μM and 1.15 μM against MGC803, MCF7 and PC3 cells, respectively [246]. Structure–activity relationships reveal that its 3,4,5-trimethoxyphenyl ring exerts a crucial role for the antiproliferative activity against all cancer cell lines. Compound **10**, a novel tertiary amide derivative, activated NEDDylation in a concentration-dependent manner through the direct interaction with APPBP1 in gastric cancer MGC803 cells. Moreover, it induces the degradation of c-IAP1 and the down-regulation of XIAP via the activation of NEDDylation (Fig. 8B). Compound **10** induces cell morphology changes, causes apoptosis and arrests cell cycle in G2/M phase against MGC803 cells. It increases expression levels of Fasl, FADD, cleaved caspase 8/9, PIDD and Bax and decreases expression levels of pro-caspase 2 and Bcl-2 in a concentration-dependent manner against MGC803 cells. Compound **10** at 50 mg/kg obviously suppresses tumor growth in MGC803 xenograft models via the activation of NAE-UBC12-Cullin1 NEDDylation. Therefore, NAE agonists may regulate the proliferation, apoptosis and cell cycle of tumor cells through the promotion of NEDDylation, and NAE agonists can be one of the future research directions of NAE-targeted drugs for cancer therapy [247, 248].

### Non-covalent NAE inhibitors

So far, many reported NAE inhibitors inhibit the activity of NAE through covalent binding [121]. All these covalent NAE inhibitors contain a sulfamate group to enable the formation of NEDD8-compound covalent adducts with the carboxyl group of NEDD8. Although two covalent NAE inhibitors MLN4924 and TAS4464 entered into clinical trials for cancer therapy, there are no non-covalent NAE inhibitors that have entered the clinical stage. Natural product **11** and **12** (Fig. 8C) as non-covalent NAE inhibitors exhibit anti-prostate cancer effects via the degradation of Skp2, and natural product analogue **13** and natural product **14** are also identified as non-covalent NAE inhibitors by enzymatic and cellular assays [249–252]. A rhodium(III) complex **15** targeting NAE by a non-covalent binding model displays potential anti-inflammatory activity in vivo [82]. Approved drugs **16** (candesartan cilexetic), **17** (mitoxantrone) and **18** (piperacillin) are discovered as non-covalent NAE inhibitors through the drug repositioning strategy [33, 253, 254]. *N*-heterocycles **19**, **20** and **21** are discovered as non-covalent NAE inhibitors through the virtual screening strategy, and show the promising antitumor activity [255–257]. A indole analogue **22** is synthesized by us that induces apoptosis and cell cycle arrest at G2/M phase via suppression of NEDDylation and MAPK pathways [258]. Molecular docking studies reveal that non-covalent NAE inhibitors can block the interaction of NAE-NEDD8-ATP by targeting the ATP binding site of NAE. Most of reported non-covalent NAE inhibitors have not been evaluated for their antitumor effects at the animal level due to moderate antiproliferative activity against cell lines. Some inhibitors lack sufficient experiments (e.g., cellular thermal shift, co-immunoprecipitation, biolayer interferometry, isothermal titration calorimetry, or protein pull-down assays) to investigate their on-target effects and the selectivity against NAE. Therefore, there is great scope to develop non-covalent NAE inhibitors for the treatment of cancer.

### Application for other diseases beyond cancer

Currently, MLN4924 is the most reported NAE inhibitor and it has potential therapeutic effects for other diseases besides cancer (Fig. 8D). Interleukin-17A (IL-17A) plays critical roles in inflammatory diseases and MLN4924 can suppress IL-17A-induced pulmonary inflammation in vivo through the inhibition of ACT1-mediated signaling [259]. NEDDylation is overactivated after ischemic stroke and MLN4924 is a therapeutic candidate for ischemic stroke by maintaining the integrity of blood–brain barrier and reducing neutrophil extravasation [260]. MLN4924 displays potent cardioprotective effects against myocardial ischemia–reperfusion injury by the induction of autophagic flux and the up-regulation of Nrf2 dependent on sirt1 [261]. MLN4924 also exhibits neuroprotective effects against oxidative stress injury, and the inhibition of NEDDylation induced by MLN4924 play significant roles in various neurodegenerative diseases including Alzheimer’s disease, Parkinson’s disease, multiple sclerosis, Huntington’s disease, fragile X syndrome and Machado-Joseph disease [262]. MLN4924 activates the formation of choroidal neovascularization lesion by the up-regulation of autophagy and the suppression of hedgehog pathway [263]. Therefore, the development of NAE-targeting drugs to treat additional diseases beyond cancer is also a new direction for future research. These future research directions of NAE-targeting drugs are summarized in Fig. 8.

## Conclusions and perspectives

Since the NAE inhibitor MLN4924 was reported in 2009, researches on NAE-targeting compounds have rapidly developed in the field of medicinal chemistry. Many preclinical studies have revealed that a variety of NAE inhibitors and NAE agonists exhibit good antitumor effects in vitro and in vivo. MLN4924 and TAS4464 as covalent NAE inhibitors currently undergo clinical assessment to treat various tumors, especially hematological tumors. These findings demonstrate that NAE is a novel and effective therapeutic target for the treatment of cancers. The approaches to discover NAE inhibitors in this review provide a unique perspective to develop various NAE-targeting agents. Chemical structures, antitumor efficacy and detailed pharmacological mechanisms of reported NAE-targeting agents discussed in this review offer insights into the design of candidate compounds for clinical drug development. The overview of single medication or combined pharmacotherapy of MLN4924 and TAS4464 in clinical trials strongly encourages researchers to explore the therapeutic potential of NAE inhibitors in order to propose new treatments for cancers.

Even though preclinical studies and clinical trials have achieved many successes, some challenges and limitations associated with NAE inhibitors are arising. For patients with AML or MDS, the overall response rate for MLN4924 is only 17% (ClinicalTrials.gov Identifier: NCT00911066). Due to the structural similarity with AMP, the selectivity against NAE and the toxicity against normal cells of covalent NAE inhibitors may be limited. The clinical trial in phase I of TAS4464 was terminated due to hepatotoxicity (ClinicalTrials.gov Identifier: NCT02978235). Meanwhile, several side effects (tumor sphere formation, ciliogenesis and glycolysis) also limit the development of NAE inhibitors. Therefore, design of novel NAE-targeting agents requires greater consideration on the selectivity to maintain the balance of anticancer efficacy and safety.

At present, only two covalent NAE inhibitors enter into clinical trials and the development of non-covalent NAE inhibitors needs to be further deepened. Although the antitumor effects of NAE agonists illustrate the feasibility of NAE activation to design anticancer agents, the research of NAE agonists has just begun. Through the systematic summary of NAE-targeting agents, we found that combined pharmacotherapy, multi-target NAE inhibitors and developing other disease applications of NAE-targeting agents might also be future directions in this field. Development of NAE-targeting drugs may lead to some meaningful breakthroughs for disease therapy in the future.

## Data Availability

Not applicable.

## Abbreviations

NAE: NEDD8-activating enzyme
NEDD8: Neuronal precursor cell-expressed developmentally down-regulated protein 8
ATP: Adenosine triphosphate
APPBP1: Amyloid protein-binding protein 1
UBA3: Ubiquitin-like modifier activating enzyme 3
CRLs: Cullin-RING ligases
AAD: Active adenylation domain
IAD: Inactive adenylation domain
CC: Catalytic cysteine
UFD: Ubiquitin fold domain
AML: Acute myeloid leukemia
*T*_1/2_: Half-life
AUC: Area under the plasma concentration–time curve
Vss: Volume of distribution
CL: Clearance
*C*_max_: Peak concentration
IKB-α: I-kappa-B-alpha
AMP: Adenosine 5′-monophosphate
TRAIL: TNF-related apoptosis-inducing ligand
HNSCC: Head and neck squamous cell carcinoma
TCA: Tricarboxylic acid cycle
OXPHOS: Oxidative phosphorylation system
TME: Tumor microenvironment
NF-κB: Nuclear factor κB
MM: Multiple myeloma
HM: Hematologic malignancies
HL: Hodgkin lymphoma
MS: Myelodysplastic syndromes
ALL: Acute lymphoblastic leukemia
CLL: Chronic lymphocytic leukemia
CMML: Chronic myelomonocytic leukemia
MPN: Myeloproliferative neoplasm
MDS: Myelodysplastic syndromes
PTEN: Chromosome ten
AKT1: Protein kinase B
PKM2: Pyruvate kinase isoform M2
IL-17A: Interleukin-17A
